# Effective Delivery of Hypertrophic miRNA Inhibitor by Cholesterol‐Containing Nanocarriers for Preventing Pressure Overload Induced Cardiac Hypertrophy

**DOI:** 10.1002/advs.201900023

**Published:** 2019-04-06

**Authors:** Ying Zhi, Chen Xu, Dandan Sui, Jie Du, Fu‐Jian Xu, Yulin Li

**Affiliations:** ^1^ Beijing Anzhen Hospital Capital Medical University The Key Laboratory of Remodeling‐Related Cardiovascular Diseases Ministry of Education Beijing Institute of Heart Lung and Blood Vessel Diseases Beijing 100029 China; ^2^ Key Lab of Biomedical Materials of Natural Macromolecules (Beijing University of Chemical Technology) Ministry of Education Beijing Laboratory of Biomedical Materials Beijing Advanced Innovation Center for Soft Matter Science and Engineering Beijing University of Chemical Technology Beijing 100029 China

**Keywords:** cardiac hypertrophy, cardiomyocyte transfection, gene therapy, miRNA delivery, nanocarriers

## Abstract

Persistent cardiac hypertrophy causes heart failure and sudden death. Gene therapy is a promising intervention for this disease, but is limited by the lack of effective delivery systems. Herein, it is reported that CHO‐PGEA (cholesterol (CHO)‐terminated ethanolamine‐aminated poly(glycidyl methacrylate)) can efficiently condense small RNAs into nanosystems for preventing cardiac hypertrophy. CHO‐PGEA contains two features: 1) lipophilic cholesterol groups enhance transfection efficiency in cardiomyocytes, 2) abundant hydrophilic hydroxyl groups benefit biocompatibility. miR‐182, which is known to downregulate forkhead box O3, is selected as an intervention target and can be blocked by synthetic small RNA inhibitor of miR‐182 (miR‐182‐in). CHO‐PGEA can efficiently deliver miR‐182‐in into hearts. In the mice with aortic coarctation, CHO‐PEGA/miR‐182‐in significantly suppresses cardiac hypertrophy without organ injury. This work demonstrates that CHO‐PGEA/miRNA nanosystems are very promising for RNA‐based therapeutics to treat heart diseases.

## 1 Introduction

Cardiovascular disease (CVD) remains the major cause of morbidity and mortality. Cardiac hypertrophy, characterized by an enlargement of cardiomyocytes (CMs) and heart mass, is a central process of hypertensive heart disease, myocardial infarction, hypertrophic cardiomyopathy, and other CVDs.^1^ Persistent cardiac hypertrophy leads to heart failure (HF) as well as sudden death.^2^ Currently, clinical therapeutics are primarily to decrease cardiac pressure or volume overload and/or block adrenergic receptors.^3^ However, those therapies do not achieve the desired effect. As an important antihypertrophy transcription factor, forkhead box O3 (FOXO3) could suppress prohypertrophic calcineurin/nuclear factor of activated T‐cells (NAFT) signaling pathway and transcriptionally activate catalase and atrogin‐1, which is downregulated in hypertrophic heart.^4^, ^5^, ^6^, ^7^ It was reported that miR‐182 could target FOXO3 3′ untranslated region (3′ UTR) and suppresses its expression, which is upregulated in CMs of hypertrophic mouse heart or heart tissues of patients with heart failure.^8^, ^9^ It is interesting to evaluate whether the manipulation of specific miRNAs such as miR‐182 would yield a therapeutic strategy for hypertrophy and heart failure.

A successful miRNA‐based gene therapy requires not only a specific gene target, but also a safe and effective delivery system.^10^ miRNAs are highly susceptible to degradation by ribonuclease (RNase) in serum, thus protecting miRNAs from degradation is one of the necessary requirements for gene delivery.^11^ Furthermore, CMs, which are extremely associated with cardiac hypertrophy, are difficult to be transfected by gene vectors.^12^, ^13^ Thus, improving the transfection efficiency of vectors in CMs is a challenge to be overcome. As the major type of nonviral vectors, polycations are positively charged and can condense the negatively charged RNAs or DNAs into nanocomplexes through electro‐interaction, thus protect them from degradation and help them to be internalized by cell.^11^ Due to their low host immunogenicity and flexibility, various of polycations have been developed for gene therapy, for example gold‐standard polyethyleneimine (PEI).^14^, ^15^ Recently, we reported cholesterol (CHO)‐terminated ethanolamine‐aminated poly(glycidyl methacrylate) (CHO‐PGEA) for efficient plasmid DNA delivery.^16^

**Figure 1:**
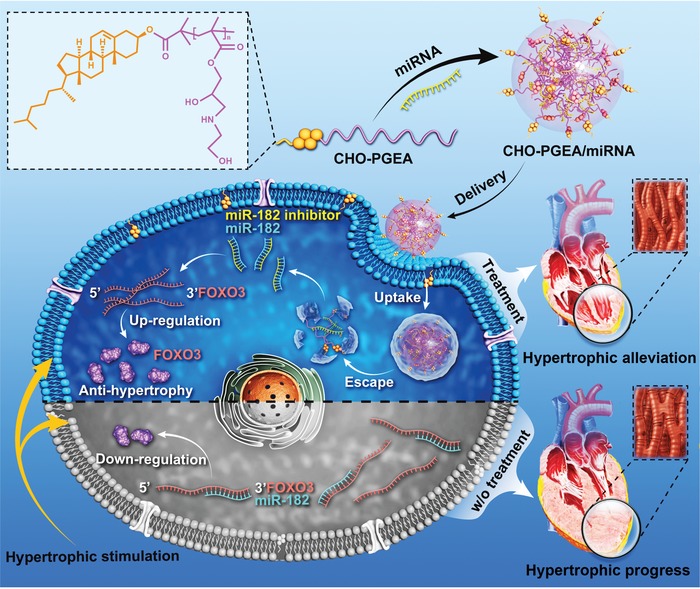
Schematic diagram illustrating the preparation of CHO‐PGEA/miRNA complexes and the resultant gene therapy in hypertrophic cardiomyocytes (CMs).

Due to its similar components to cell membrane, CHO‐PGEA could increase the efficiency of cellular uptake.^16^ CMs are characterized by transverse tubules (TT) which possess rich cholesterol species.^17^ TT, which is formed by invaginated CM membrane, increases the ratio of surface area to volume.^18^ In this study, CHO‐PGEA/miRNA nanosystems were proposed to deliver synthetic small RNA inhibitor of miR‐182 (miR‐182‐in) (**Figure** **1**). MiR‐182‐in was delivered into CMs to specifically bind miR‐182 through base pairing, preventing deterioration of cardiac hypertrophy. A series of in vitro and in vivo experiments were carried out to investigate whether CHO‐PGEA/miR‐182‐in would contribute to the prevention of cardiac hypertrophy caused by pressure overload.

## 2 Results and Discussions

### 2.1 Preparation and Characterization of CHO‐PGEA/miRNA

For the preparation of CHO‐PGEA/miRNA nanoparticles, CHO‐PGEA consisting of cholesterol and low‐toxic PGEA was prepared based on CHO‐terminated poly(glycidyl methacrylate) (CHO‐PGMA, Mn = 9.25 × 10^3^ g mol^−1^) as reported before.^16^ Effective capacity to condense nucleic acid is prerequisite for a good gene vector, which was evaluated by agarose gel electrophoresis, dynamic light scattering, ζ‐potential and atomic force microscope (AFM) imaging. Gold‐standard PEI (25 kDa) was also tested for comparison.^19^

**Figure 2:**
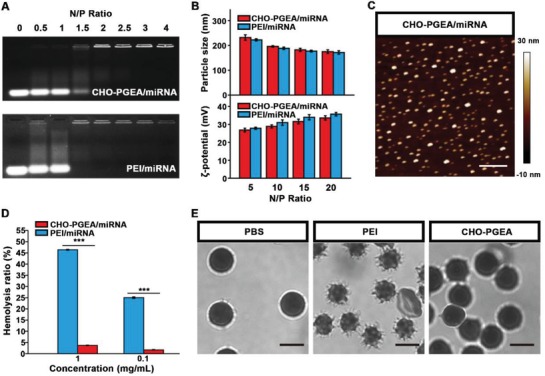
A) Electrophoretic mobility retardation assay of polycation/miRNA complexes at various N/P ratios. B) Particle size and ζ‐potential of the polycation/miRNA complexes at various N/P ratios. C) AFM images of polycation/miRNA complexes at the typical N/P ratio of 10 (scale bar: 500 nm). D) Qualification of hemolysis ratio and E) images of red blood cells (RBCs) treated with PEI and CHO‐PGEA at the concentration of 1 mg mL^−1^ (scale bar: 5 µm), where PBS was used as the control. ***P < 0.001; Data are from 3 independent experiments.

As shown in **Figure** **2**A, both of CHO‐PGEA and PEI could completely condense miRNA when the N/P ratio reached to 2. Also, the particle sizes and ζ‐potentials of CHO‐PGEA/miRNA and PEI/miRNA complexes at various N/P ratios were measured by dynamic light scattering (DLS) (Figure 2B). The particle sizes of all the complexes ranged from 150–250 nm at various N/P ratios with good distribution (Table S1, Supporting Information). Also, all the complexes had positive ζ‐potentials ranging from 25–40 mV. Positive charges could benefit the complexes to adhere to negatively‐charged cell membranes, finally resulting in the facilitation of cellular uptake. The above results illustrated that miRNA could be effectively condensed by CHO‐PGEA and PEI. In addition, AFM was used to confirm the condensation ability of CHO‐PGEA. CHO‐PGEA/miRNA complexes at the N/P ratio of 10 were selected to be imaged as shown in Figure 2C. CHO‐PGEA/miRNA complexes were in the regulated nanospheres.

### 2.2 Protein Absorption, Hemolysis, and In Vitro Cytotoxicity Assay

Polycationic complexes are generally not stable in blood circulation because negatively‐charged biomacromolecules such as human serum albumin would weaken the surface potential of complexes, leading to large aggregation. In addition, polycations might cause thrombosis through hemolytic reaction. Various CHO‐PGEA/miRNA complexes were treated with bovine serum albumin (BSA) to evaluate the antiprotein adsorption capacity (Figure S1, Supporting Information). With the treatment by BSA, PEI/miRNA complexes rapidly adsorbed protein and only less than 5% of pristine free BSA remained after 1 h. However, CHO‐PGEA/miRNA complex exhibited better antiprotein adsorption capacity. Even after 1 h incubation, there was still about 47% of free BSA remained.

The hemolysis assays of PEI and CHO‐PGEA were also measured with red blood cells of C57BL/6J mice. As shown in Figure 2D, at the concentration of 1 mg mL^−1^, the hemolysis ratios of CHO‐PGEA was below 2%, which was significantly lower than 25% of PEI. Moreover, the images of red blood cells (RBCs) were taken by confocal laser scanning microscopy (CLSM) to evaluate the structural integrity of RBCs, illustrating that CHO‐PGEA would not cause structural damages to RBCs (Figure 2E).

Low cytotoxicity was also essential for delivery systems. As shown in Figure S2A (Supporting Information), in comparison with those of PEI/miRNA, the cytotoxicities of CHO‐PGEA/miRNA complexes were much lower at various N/P ratios in H9C2 cells (embryonic rat cardiac myocytes line). The above excellent abilities of CHO‐PGEA were probably due to the amounts of flexible hydroxyl groups of PGEAs which can effectively balance the surface potential of complexes and reduce the deleterious effects of excess positive charges.^11^, ^19^, ^20^, ^21^ These results indicated that CHO‐PGEA/miRNA nanosystems are probably suitable for in vivo delivery of nucleic acids.

### 2.3 Cellular Internalization in CMs and Hearts

**Figure 3:**
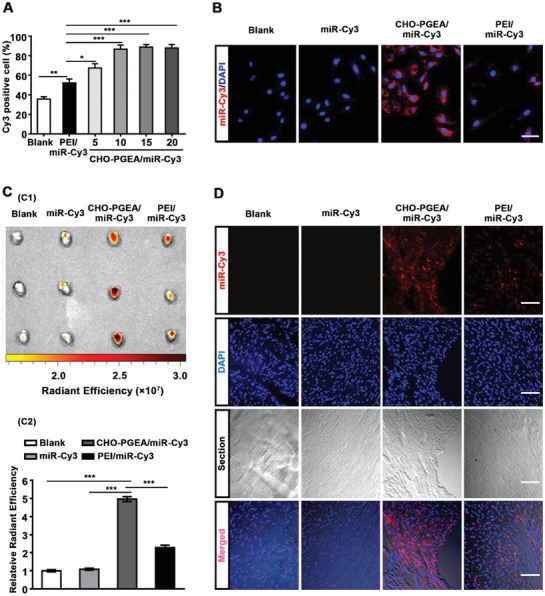
Cellular internalization of polycation/miRNA complexes in CMs or heart. A) Cellular internalization assay by MD where PEI/miR‐Cy3 was performed at the N/P ratio of 10. B) Confocal fluorescence images of H9C2 cells treated with miR‐Cy3 or polycation/miR‐Cy3(red) at the N/P ratio of 10 (scale bar: 50 µm). C) Image and relative radiant efficiency of mice hearts treated with saline, free miR‐Cy3, PEI/miR‐Cy3 or CHO‐PGEA/miR‐Cy3. D) Fluorescence images of heart section in four groups (scale bar: 100 μm). *P < 0.05, **P < 0.01, and ***P < 0.001; Data are from 3 independent experiments.

Cellular internalization is a critical factor of the successful gene therapy. In general, PEI/miRNA complex exhibits the best gene transfection effect at the N/P ratio of 10,^16^, ^19^ which was used as a positive control. MiR‐Cy3 is a negative control miRNA labeled with fluorescent dye Cy3 which is used for the studies of in vitro and in vivo cellular uptake. The cellular uptake assays of polycation/miR‐Cy3 complexes were performed by high‐content screening (HCS), showing that all the CHO‐PGEA/miR‐Cy3 complexes exhibited better cellular uptake ability compared with free miR‐Cy3 and PEI/miR‐Cy3 complexes (**Figure** **3**A).

The percentage of positive cells treated with CHO‐PGEA/miR‐Cy3 complexes was more than 67% at the low N/P ratio. Especially when the N/P ratio was greater than 10, the percentages of positive cells were about 87%. CHO‐PGEA/miRNA complex at the N/P ratio of 10 was selected as the suitable recipe for gene delivery based on the analysis of cytotoxicity and cell internalization. At the N/P of 10, the average fluorescent intensities of the positive cells treated with CHO‐PGEA/miR‐Cy3 complexes was 1.5 times than those treated with PEI/miR‐Cy3 complexes (Figure S2B, Supporting Information). CLSM was also used to evaluate the cellular uptake of polycation/miR‐Cy3 complexes in H9C2 cells (Figure 3B). The nucleus was stained by 4′,6‐diamidino‐2‐phenylindole (DAPI) in blue, and the Cy3‐labeled miRNA showed red. Compared to free miR‐Cy3 and PEI/miR‐Cy3 complexes, more CHO‐PGEA/miR‐Cy3 complexes could be observed in H9C2 cells, demonstrating CHO‐PGEA owned higher cellular uptake ability. From the data above, CHO‐PGEA could efficiently transport miRNAs into CMs. In addition, our pervious study had shown that CHO‐PGEA had better capability for cellular internalization than the counterpart without cholesterol modification,^16^ illustrating the importance of cholesterol group in intracellular gene delivery.

An effective delivery system for gene therapy should ultimately work in vivo. To evaluate the delivery efficiency of CHO‐PGEA in mice heart, we injected the free miRNA‐Cy3, PEI/miR‐Cy3 and CHO‐PGEA/miR‐Cy3 into the wild‐type (WT) mice via intravenous injection. In vivo fluorescence imaging of small animals showed that the fluorescence emitted from the heart treated with free miR‐Cy3 was weak (Figure 3C1). Whereas the fluorescence intensity of heart tissues was significantly increased after the injection of CHO‐PGEA/miR‐Cy3, even much stronger than that in mice heart treated with PEI/miR‐Cy3. As shown in Figure 3C2, the cardiac fluorescence emitted by miR‐Cy3 was collected and quantified by relative radiant efficiency. The result of radiant efficiency was increased by more than twofold in the CHO‐PGEA/miRNA‐Cy3 group compared with the PEI/miRNA‐Cy3 group. Moreover, miR‐Cy3 entering the heart was observed by fluorescence microscopy in heart tissue sections (Figure 3D). Compared with other groups, the accumulation of miR‐Cy3 was obviously increased in heart tissues of CHO‐PGEA/miR‐Cy3 group. These data indicated that both of CHO‐PGEA and PEI were able to protect miRNA from the degradation of RNase in blood and transport miRNA into hearts, while the delivery efficiency of CHO‐PGEA was better than PEI in vivo. The better delivery efficiency of CHO‐PGEA was probably due to the cholesterol group which makes complexes easier to dissolve with lipids on cell membranes and access to CMs. In addition, the above better antiprotein adsorption capacity of CHO‐PGEA may also contribute to the delivery efficiency.

### 2.4 Delivery of miR‐182 and its Biological Performance

**Figure 4:**
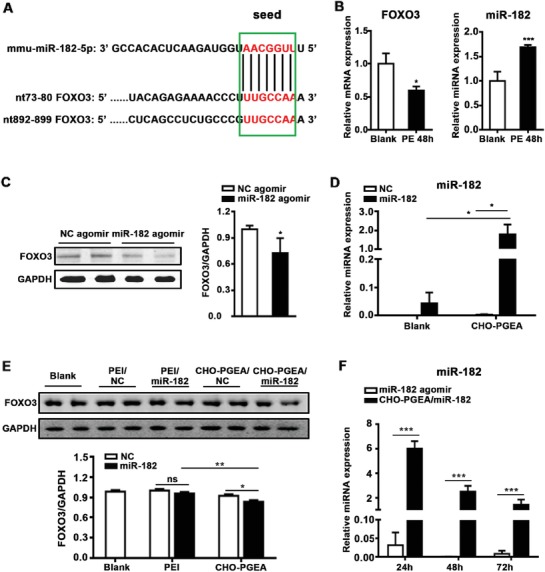
miRNA was effectively delivered into CMs by CHO‐PGEA and preform biology function. A) Alignment of mmu‐miR‐182 with putative 3′UTR target sites: 2 sites in FOXO3. miR‐182 seed sequence and the corresponding target sites were indicated in green box. Complementary bases were shown in red color. B) qRT‐PCR shows relative expression of miR‐182 and FOXO3 mRNA in cardiomyocytes after PE stimulation for 48 h. C) Representative FOXO3 expression after overexpressing miR‐182 in heart by western blot (WB). D) qRT‐PCR shows relative expression levels of miR‐182 in H9C2 cells treated with CHO‐PGEA/miR‐182 complexes after 24 h. E) Protein level of FOXO3 in H9C2 cells administered with polycation/miRNA. F) Relative expression of miR‐182 in mouse hearts after intravenous (IV) injection of CHO‐PGEA/miR‐182 complexes (5 nmol) or miR‐182 agomir (5 nmol), (*n* = 3 per group). *P < 0.05, **P < 0.01, ***P < 0.001 by Student's *t*‐test or one‐way ANOVO.

MicroRNA promotes the degradation of mRNA or inhibits translation of protein by binding to the 3 ′UTR region of the target gene.^22^, ^23^, ^24^ MiR‐182 derived from CMs may downregulate the level of FOXO3.^25^, ^26^ As shown in **Figure** **4**, we validated the interaction between miR‐182 and FOXO3. Bioinformatics (TargetScan database) predicts two binding sites of miR‐182 in FOXO3 mRNA 3′UTR region (Figure 4A). We further examined the expression of FOXO3 and miR‐182 in isolated neonatal mouse primary cardiomyocytes after stimulating phenylephrine (PE, hypertrophic stimulation) for 48 h. Before detecting RNA expression, we confirmed that PE‐induced CMs hypertrophy was successful through myocardial cytoskeletal protein (α‐actinin) staining and hypertrophic gene (atrial natriuretic peptide (ANP)) detection (Figure S3, Supporting Information). The result of quantitative real‐time polymerase chain reaction (qRT‐PCR) showed a decrease of FOXO3 mRNA level, accompanied by an increase in miR‐182 levels (Figure 4B). Moreover, the protein level of FOXO3 was substantially decreased by 27.5% in WT mice administered with miR‐182 agomir (Figure 4C), indicating that miR‐182 was a negative‐regulatory factor of FOXO3.

A successful delivery system not only requires efficient delivery of miRNA into cells, but also ensures that its biological function could be not affected. Therefore, miR‐182 was used to investigate the transfection efficiency of CHO‐PGEA as well as its impact on miRNA function. The expression of miR‐182 in H9C2 cells, which were separately treated with free negative control miRNA (miR‐NC), free miR‐182, CHO‐PGEA/negative control miRNA (CHO‐PGEA/miR‐NC) or CHO‐PGEA/miR‐182, was examined by qRT‐PCR. Compared with the blank control (including free miR‐NC or miR‐182 group) and CHO‐PGEA/miR‐NC groups, the expression of miR‐182 mediated by CHO‐PGEA occurred an obvious increase, which was relative to the internal reference U6 (Figure 4D). Furthermore, to confirm that miR‐182 delivered by CHO‐PGEA could exert normal biological functions, we detected the protein levels of FOXO3 in H9C2 cells transfected with free miRNAs, PEI/miRNAs or CHO‐PGEA/miRNAs (miRNAs included miR‐182 and miR‐NC, respectively) for 24 h by western blot. There was no significant difference between the PEI/miR‐NC and PEI/miR‐182 groups, suggesting that PEI affected the function of miR‐182 which might be due to the insufficient release of miR‐182 mediated by PEI in CMs. The protein level of FOXO3 was significantly decreased in the CHO‐PGEA/miR‐182 group compared with CHO‐PGEA/miR‐NC (Figure 4E), indicating that CHO‐PGEA was promising in delivering miRNA and exerting its biological function.

Next, we compared the delivery efficiency of miRNA through CHO‐PEGA and commercial reagents (including agomir and antagomir) in vivo. Since the expression of antagomir and inhibitor is not easy to be evaluated, we compared the effects between CHO‐PGEA/miR‐182 and miR‐182 agomir by detecting the expression of miR‐182 by qRT‐PCR. At the same dose (5 nmol), the expression of miR‐182 in the CHO‐PGEA/miR‐182 group was substantially higher than that in the miR‐182 agomir group, indicating that CHO‐PGEA/miR‐182 was more efficient than miR‐182 agomir in mouse heart. Also, we found that CHO‐PGEA/miR‐182 complex still could increase expression of miR‐182 in heart tissues after 72 h for a single injection, while miR‐182 agomir group returns to the basic level at 48 h after injection (Figure 4F). CHO‐PGEA exhibited more advantages than commercial agomir including better delivery efficiency and longer time to maintain high expression of miRNA in heart. Hypertrophy is a long‐term chronic pathological process, hence its treatment is also continuous. The good performances of CHO‐PGEA in transfection efficiency and maintenance time in vivo reduced the dosage and the dosing frequency of exogenously miRNA, thereby also probably reducing the side effects to other organs due to systemic administration. In addition, the reduction of dosing frequency could improve patient compliance. CHO‐PGEA would be beneficial for gene therapy in hypertrophic cardiomyopathy or HF.

### 2.5 Therapy of Cardiac Hypertrophy by CHO‐PGEA/miR‐182‐in

**Figure 5:**
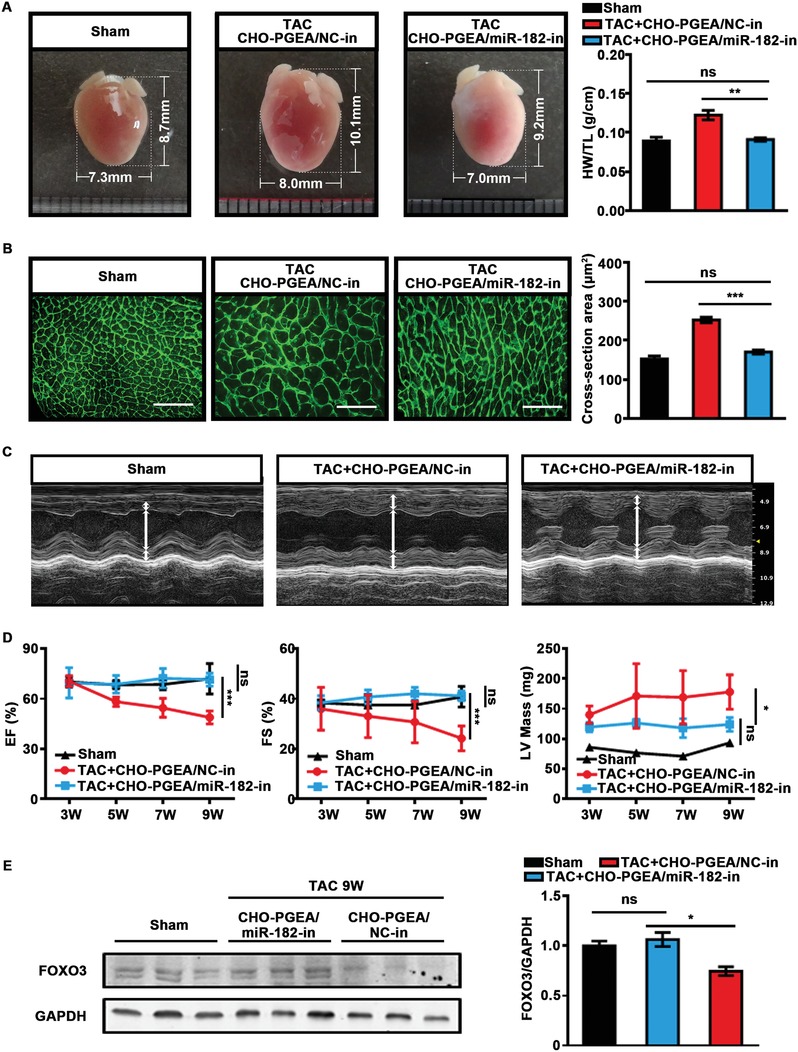
CHO‐PGEA/miR‐182‐in complex prevents hypertrophy. A) Gross phenotypic pattern captured by digital camera and heart weight (HW)/tibia length (TL) ratio at the end of treatment. B) Images of wheat germ agglutinin (WGA) stained hearts and quantification of myocyte cross section areas (scale bar: 50 µm). C) Left ventricle (LV) M‐mode images and D) echocardiographic data of mice hearts administered with CHO‐PGEA/miRNA‐in complexes from the 3rd week to the 9th week after TAC surgery, including ejection fraction (EF), FS, and LV mass. E) FOXO3 protein levels in mice heart after TAC by WB. *P < 0.05, **P < 0.01, and ***P < 0.001 (*n* = 5 per group).

In view of high transfection efficiency of CHO‐PGEA/miRNA, we hypothesized that CHO‐PGEA also could deliver miR‐182‐in (antisense oligonucleotide of miR‐182) into heart, then improve the pressure overload‐induced cardiac hypertrophy. The C57BL/6J mice were established with transversal aortic constriction (TAC) animal models for 9 weeks, leading to cardiac hypertrophy. From the 3rd to 9th week post‐TAC surgery, the mice were administered with CHO‐PGEA/miR‐182‐in complexes (containing 5 nmol miR‐182‐in) and its negative control (CHO‐PGEA/miR‐NC‐in, containing 5 nmol miR‐NC‐in) by intravenous injection. According to the previous data (Figure 4F), the frequency of administration of CHO‐PGEA/miRNA inhibitor complexes was set to one time per 3 days to ensure that miR‐182‐in could maintain high levels and fully inhibit the function of miR‐182 in mice heart. Compared with the CHO‐PGEA/miR‐NC‐in group, heart size and heart weight (correcting by the tibia length) significantly decreased in the CHO‐PGEA/miR‐182‐in group at 9 weeks after TAC surgery (**Figure** **5**A). Wheat germ agglutinin (WGA) staining of heart was used to assess the size of CM by staining cardiomyocyte membrane. The increase in the cross‐section area of CMs in the CHO‐PGEA/miR‐182‐in group was lower than in the CHO‐PGEA/miR‐NC‐in group (Figure 5B). Moreover, after the treatment with CHO‐PGEA/miR‐182‐in, the left ventricle (LV) mass in mice was maintained at about 120 mg, while the LV mass was gradually increasing in mice treated with CHO‐PGEA/miR‐NC‐in (Figure 5D). At the 9th week after TAC surgery, the LV mass of CHO‐PGEA/miR‐NC‐in group was significantly higher than that of the CHO‐PGEA/miR‐182‐in group (increased by about 45%). These data indicated that CHO‐PGEA/miR‐182‐in could effectively suppress cardiac hypertrophy.

Meanwhile, with the prolongation of the TAC surgery time, both the cardiac ejection fraction (EF) and the fractional shortening (FS), which were confirmed by echocardiography analysis, gradually decreased in the CHO‐PGEA/miR‐NC‐in group, but maintained at a relatively normal level in the CHO‐PGEA/miR‐182‐in group (Figure 5C,D). Persistent pathological cardiac hypertrophy leads to heart entering the decompensation phase, causing a decrease in cardiac function. Our results demonstrated that CHO‐PGEA/miR‐182‐in complex could block cardiac hypertrophy induced by pressure overload, thus improve cardiac function. The protein level of FOXO3 in hearts of mice in the CHO‐PGEA/miR‐182‐in group was higher than that in the negative control group (Figure 5E). Mechanically, CHO‐PGEA/miR‐182‐in restored the protein level of FOXO3 and treated hypertrophy.

**Figure 6:**
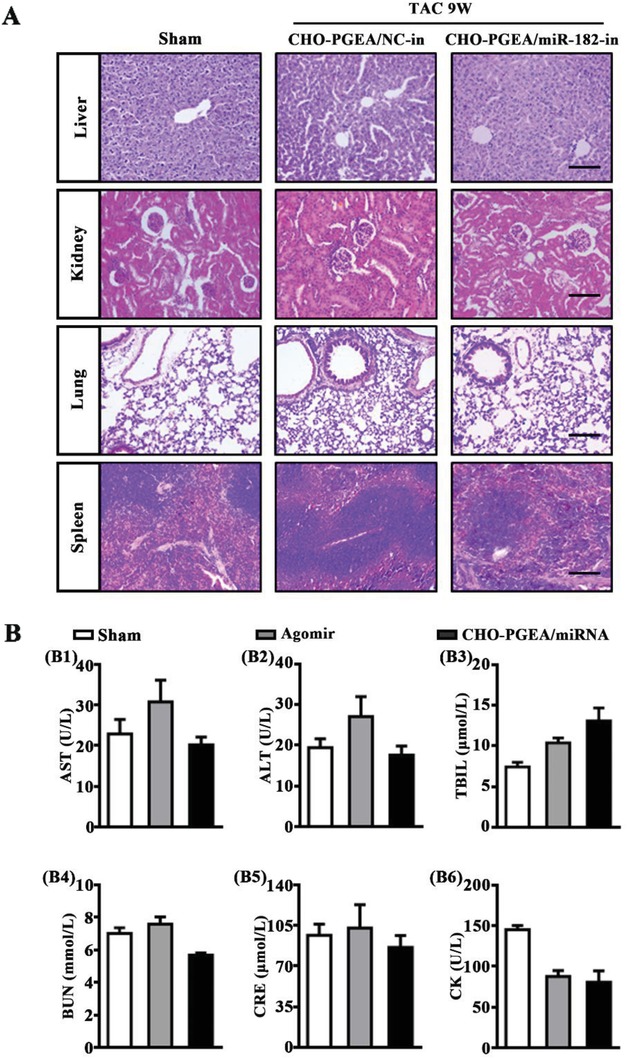
CHO‐PGEA/miRNA complexes toxic effect on organs. A) Representative photographs of H&E staining of the liver, kidney, lung, and spleen in mice treated with CHO‐PGEA/miRNA‐in after TAC 9 weeks (bar: 100 µm). B) Plasma biochemical measurement for aspartate transaminase (AST), alanine transaminase (ALT), total bilirubin (TBIL), blood urea nitrogen (BUN), creatinine (CRE), and creatine kinase (CK) in mice with saline, agomir or CHO‐PGEA/miRNA for 24 h (*n* = 3 per group).

In addition to low cytotoxicity in vitro (Figure S2A, Supporting Information), it was also important to study if there is also a low organ toxicity of CHO‐PGEA/miRNA. Finally, hematoxylin and eosin (H&E) staining of pathological sections of liver, kidney, lung, and spleen tissues did not show significant changes in the morphologies of the tissues after administration, demonstrating the safety of long‐term administration to other organs (**Figure** **6**A). In addition, aspartate aminotransferase (AST), alanine aminotransferase (ALT), total bilirubin (TBIL), blood urea nitrogen (BUN), and creatinine (CRE) were maintained at normal levels in mice plasma after injection of CHO‐PGEA/miRNA complexes and miRNA agomir, indicating that acute injury did not occur in liver and kidney after injection of CHO‐PGEA/miRNA for 24 h (Figure 6B). Creatine kinase (CK) remained at normal level, suggesting that there is no cardiac toxicity after short‐term administration (Figure 6B). Taken together, our findings delineated that inhibition of miR‐182 with CHO‐PGEA indeed avoids pathological cardiac hypertrophy induced by pressure overload.

Our finding elucidated that CHO‐PGEA has potential significant application value in cardiac hypertrophy‐related diseases. The functional analyses of dysregulated miRNAs, which are increased or decreased in hypertrophic heart and/or CMs, have demonstrated that miRNAs may exert either positive or negative regulatory effects on cardiac hypertrophic pathways.^27^ The roles of miR‐1, miR‐133 or miR26b in hypertrophy are shown to target single or multiple prohypertrophic genes.^28^, ^29^, ^30^ On the other hand, the prohypertrophic miRNAs, such as miR‐199a, miR‐208a/b, miR‐21 or miR‐22, play roles by inhibiting the expression of antihypertrophic genes.^28^, ^31^, ^32^, ^33^, ^34^ These miRNAs are also suitable for hypertrophic gene therapy with CHO‐PGEA as a delivery vehicle.

## 3 Conclusions

In summary, CHO‐PGEA successfully transported miR‐182‐in into cardiomyocytes or heart tissues, which was served as a treatment strategy for cardiac hypertrophy. CHO‐PGEA condensed miRNA, exhibited excellent delivery efficiency, and successfully released miR‐182‐in to restore the level of FOXO3 in heart induced by pressure overload. After the treatment of CHO‐PGEA/miR‐182‐in, the process of cardiac hypertrophy was effectively inhibited in pressure overload‐induced mice. In addition, for other organs, such as liver or kidney, CHO‐PGEA‐based therapy caused neither acute functional damages, nor structural changes. This study elucidated that CHO‐PGEA/miRNA could be severed as one effective and promising nanosystem for the therapy of heart diseases.

## 4 Experimental Section

*Materials*: Branched polyethyleneimine (Mw ≈25 kDa), cholesterol (95%), streptomycin, penicillin and 4′,6‐diamidino‐2‐phenylindole, 3‐(4,5‐dimethylthiazol‐2yl)‐2,5‐diphenyl tetrazolium bromide (MTT), and fluorescein isothiocyante (FITC) conjugated wheat germ agglutinin were bought from Sigma‐Aldrich Chemical Co. (St. Louis, MO, USA). GMA was used after removal of the inhibitors (Sigma‐Aldrich). Phosphate buffered saline (PBS) and Dulbecco's modified Eagle medium (DMEM) were obtained from Thermo Scientific HyClone Co. (St. Utah, Waltham, MA). Trypsin and fetal bovine serum (FBS) were purchased from Gibco Co. Trizol lysis buffer was obtained from Invitrogen Co. The synthetic microRNA‐negative control (miR‐NC, scramble miRNA molecules), microRNA‐negative control inhibitor (miR‐NC‐in, antisense oligonucleotide of scramble miRNA molecules), scramble control labeled with the fluorescent dye Cy3 (miR‐Cy3), miR‐182, and miR‐182‐in (antisense oligonucleotide of miR‐182) were purchased from RIBBIO Co. Ltd (Guangzhou, China). The anti‐FOXO3 and anti glyceraldehyde‐3‐phosphate phosphate dehydrogenase (anti‐GAPDH) were obtained from Cell Signaling Technology (CST, Cambridge, MA) and Zhongshan Golden Bridge (Beijing, China), respectively.

*Synthesis of CHO‐PGEA*: The proposed CHO‐PGEA was prepared via the combination of atom transfer radical polymerization (ATRP) of GMA and ring‐opening reaction of EA. CHO‐terminated poly(glycidyl methacrylate) (CHO‐PGMA, Mn = 9.25 × 10^3^ g mol^−1^ with polydispersity index of 1.3) and relative CHO‐PGEA were prepared as reported before.^16^

*Preparation of Polycation/miRNA Complexes*: N/P ratios of different polycation/miRNA complexes were molar ratios of nitrogen (N) in polycation to phosphate (P) in miRNA. All the measurements to construct complexes were described earlier.^11^

*Physicochemical Characterization*: Detailed measurements about dynamic light scattering, atomic force microscopy, gel electrophoresis, and protein absorption of polycation/miRNA complexes were described in the earlier work.^11^, ^20^

*Hemolysis Assay*: Hemolysis assays of polycations were performed with red blood cells of C57BL/6J mice. Mice blood were collected through cardiac puncture and suspended in PBS. RBCs were separated from serum and washed with PBS for three times until the supernatant was clear. All the hemolysis ratios were measured according to previous work.^35^

The morphological changes of RBSs treated with deionized PBS, PEI, and CHO‐PGEA at the concentration of 1 mg mL^−1^ were observed. After the incubation at 37 °C for 4 h, each RBCs solution was centrifuged at 20 000 rpm for 20 min. Then the structures of collected RBCs were captured by confocal laser scanning microscope (Leica, Germany).

*In Vitro Cytotoxicity Assay*: The cell viability of different polycation/miRNA complexes at various N/P ratios was assessed using H9C2 cell line via 3‐(4,5‐dimethyl‐2‐thiazolyl)‐2,5‐diphenyl tetrazolium bromide (MTT) assay. H9C2 cells were previously cultured in 96‐well plates for 24 h. Then different polycation/miRNA complexes at various N/P ratios were added into the medium. 24 h later, the MTT reagent was added to each well for examination. The final absorbance was recorded at a wavelength of 570 nm via Cytation imaging reader.

*In Vitro Cellular Uptake Assay*: MiR‐Cy3 was used for cellular uptake assay. 5 × 10^4^ H9C2 cells per well were seeded in a 24‐well plate with 500 µL per well of normal medium. After 24 h incubation under normal conditions, the culture medium was replaced with fresh medium containing polycation/miR‐Cy3 complexes with 2 µg of miR‐Cy3 (prepared previously at the N/P ratios of 5 to 20) each well. 4 h later, the medium was substituted with fresh medium and cells were cultured for additional 20 h. The cultured cells were washed with PBS twice and DAPI (150 ng mL^−1^ in PBS) was used to stain the nuclei of cultured cells for 10 min. Then, the cells were visualized and quantified with high content scanner (Molecular Devices, Silicon Valley, CA) using a MataXpress software. The images of cellular uptake for some typical complexes were observed by confocal laser scanning microscope (Leica, Germany).

*Description of Animals*: 10–12 weeks old male wild type mice (C57BL/6J background) were used in this study. All mice, which were from the Chinese Academy of Medical Sciences (Beijing, China) were kept under specific‐pathogen‐free conditions at a controlled temperature in the Beijing institute of Heart Lung and Blood Vessel Diseases and were given free access to food and water. All animal studies were approved by the Animal Care and Utilization Committee of Capital Medical University and performed in accordance to the Guide for the Care and Use of Laboratory Animals (NIH Publication No. 85‐23, revised in 2011). Although no a priori power analysis was performed, a limited number of animals were used in order to respect animal welfare regulations. Experimental numbers were based on previous experience with the used animal models. No specific randomization techniques were used. Mice were euthanized by pentobarbital (200 mg kg^−1^, intraperitoneal injection) at the end of treatment.

*In Vivo Transfection Efficiency Assay*: A total of twenty male C57BL/6J mice were divided into four groups (one blank control group and three experimental groups). In control group, mice were injected with saline, while the mice in other groups were treated with miR‐Cy3, PEI/miR‐Cy3 complexes or CHO‐PGEA/miR‐Cy3 separately via eye canthus intravenous (IV) injection. The complexes were prepared in the N/P ratio of 10 and each injection dose of the complex solution containing 5 nmol miR‐Cy3 was 100 µL. After 1 h, the fluorescence signals of polycation/miR‐Cy3 complexes in heart were captured and analyzed using the Xenogen IVIS imaging system (USA). The mouse heart sections were observed with CLSM to detect the entry of polycation/miR‐Cy3 complexes into mouse hearts.

*Transverse Aortic Constriction*: TAC was performed as described previously.^36^ Briefly, mice were anesthetized with 1% pentobarbital (70 mg kg^−1^), and then subjected to TAC by tying a 5‐0 nylon suture ligature against a 27‐gauge blunt needle. The blood flow velocity of the aortic arch contraction was maintained at 3000–4000 mm s^−1^. Mice were monitored up to 9 weeks after TAC procedure.

*Agomir Administration*: Three groups of mice were treated with miR‐182 agomir via IV injection. Each group contained 3 mice, and injection dose containing 5 nmol miR‐182 agomir was 100 µL. Three groups of mice were sacrificed at 24, 48, and 72 h after injection of agomir, respectively. Blood and PBS‐perfused heart tissues were obtained. The expression of miR‐182 in hearts of each group was detected by qRT‐PCR.

In another experiment, mice were divided into sham group (*n* = 10) and TAC group (*n* = 10). Both two groups were administered with negative control (miR‐NC) agomir (*n* = 5) or miR‐182 agomir (*n* = 5), which contained 20 nmol with 100 uL DEPC water. Mice were sacrificed at the 8th week after TAC. Hearts were used for assessing protein level of FOXO3 through western blot (WB).

*In Vivo Treatment by CHO‐PGEA/miRNA Complexes*: To assess the efficiency of CHO‐PGEA/miRNA delivery in the heart, three groups of mice were established that were sacrificed at 24, 48, and 72 h after a single injection of CHO‐PGEA/miR‐182 (5 nmol per mouse). Detection method was consistent with agomir administration. To observe the treatment effect, 20 male WT mice were divided into two main groups comprising sham group and TAC groups. In each main group, mice were treated with CHO‐PGEA/miR‐NC or CHO‐PGEA/miR‐182‐in complexes via IV injection. Each injection dose containing 5 nmol RNA was 100 uL. All the groups were started to inject at 3 weeks after TAC surgery, once every 3 days for six weeks. During the period, the cardiac function was observed by echocardiography (from the 3rd week to the 9th week after TAC, once every two weeks). All mice were sacrificed at the end of the experiment and multiple organs including heart, liver, kidney, lung, and spleen were collected.

*Transthoracic Echocardiography*: Before transthoracic echocardiography, the chest of the mice was shaved and treated with a hair‐removing cream (Veet, Reckitt Benckiser, China). Mice were anesthetized with saturated tribromoethanol. To measure global cardiac function and left ventricular mass, echocardiography was performed in mice before TAC and at 3, 5, 7, and 9 weeks after TAC. M‐mode images were acquired with a Visual Sonic 2100 high‐resolution ultrasound imaging system.^37^

*Blood Biochemical Test*: The plasma samples from mice administered with miR‐182 agomir (5 nmol) or CHO‐PGEA/miR‐182 (5 nmol) for 24 h were assayed for ALT, AST, TBIL, CRE, and BUN using an autoanalyzer (RA 1000; Technicon Instruments, NY, USA). CK activities in plasma samples were measured by commercially available kits.

*Cell Cultures*: The H9C2 cells were cultured in DMEM medium supplemented with 10% fetal bovine serum, 100 U mL^−1^ penicillin and 100 µg mL^−1^ streptomycin in tissue culture flasks at 37 °C in a humidified atmosphere of 5% CO_2_. Cardiomyocytes were isolated from 1‐to‐2‐day‐old mouse pups by enzymatic digestion and cultured in CM‐medium. 24 h later, CM was starved with FBS‐free CM‐medium for 8 h. The medium was exchanged with full DMEM medium contained PE (50 × 10^−6^ m) for 48 h.

*Histological Assessments*: Paraffin‐embedded heart tissues were sectioned at 4 µm thickness as previously described.^38^ Hematoxylin and eosin staining was used to evaluate the morphological structure of liver, lung, and spleen in mice treated with CHO‐PGEA/miRNA. For wheat germ agglutinin staining, sections were incubated with WGA in 37 °C for 1 h after serum blocking. Cardiomyocyte cross‐sectional area were determined in captured images using a Nikon Eclipse 90i microscope and analyzed by NIS‐Elements Br 4.0 software.

*Western Blot Analysis*: Western blotting was performed as previously described.^36^ In brief, protein was extracted from H9C2 cell line and left‐ventricular samples with lysis buffer containing a complete protease inhibitor cocktail (Roche, Mannheim, Germany). Following determined protein concentrations, the samples were denatured by boiling (99 °C, 5 min), separated by SDS‐PAGE (50 µg per sample) and transferred to nitrocellulose membranes. The membranes were blocked with 5% skim milk and incubated with the primary antibodies anti‐FOXO3 (1:500) and anti‐GAPDH (1:1000) at 4 °C overnight, then incubated with infrared Dye 800‐conjugated secondary antibodies (1:10 000, Rockland Immunochemicals, Inc., Gilbertsville, Pa.) for 1 h at room temperature. The images were quantified using the Odyssey infrared imaging system (LI‐COR Biosciences, Lincoln, NE).

*mRNA and miRNA Isolation and Quantification*: For quantitative real‐time PCR, total RNA was extracted by Trizol method from tissue and cells according to previously described.^39^ To assess expression of miRNA‐182, the cDNA was synthesized using the TaqMan miRNA Reverse Transcription kit (Applied Biosystems) with specific mmu‐miRNA‐182‐5p and U6 probes supplied with the TaqMan MicroRNA Assay. Then, qRT‐PCR was performed using TaqMan gene expression (Applied Biosystems) following the manufacturer's protocols. MiR‐182 expression was normalized to U6. To detect mRNA expression of FOXO3, 2 µg of total RNA were used for first‐strand cDNA synthesis with the Reverse Transcription Kit (Promega, Southampton, UK). Aliquots of 1 µL reaction mixture were amplified with 10 µL of SYBR Green PCR Master Mix and 1 µmol L^−1^ primers. Table S2 in the Supporting Information shows the primers used. Amplification was at 95 °C for 5 min, 95 °C for 45 s, and 60 °C for 1 min for each step for 45 cycles. The comparative cycle threshold method was used for the relative quantification of gene expression as previously described in the work.

*Statistical Analysis*: Physicochemical characterizations were expressed as mean ± standard deviation (SD). Other data were presented as means ± standard error of mean (SEM). Statistical analysis was performed using GraphPad Prism 6.01 (GraphPad Software Inc., San Diego, CA) and SPSS 13.0 (SPSS Inc., Chicago, IL) software. Two groups comparisons were analyzed by unpaired Student's *t*‐test. Comparison among multiple groups with one main factor was performed by one‐ or two‐way ANOVA followed by Bonferroni's post‐hoc test. The assumptions of normality were checked using Shapiro–Wilks test and equal variance was checked using Levene's test; both were satisfied. In all tests, statistical significance was set at *P* < 0.05. All experiments were repeated at least three times.

## Conflict of Interest

The authors declare no conflict of interest.

## Supporting information

Supplementary
