# Supplementary material for: Effective Delivery of Hypertrophic miRNA Inhibitor by Cholesterol‐Containing Nanocarriers for Preventing Pressure Overload Induced Cardiac Hypertrophy
Source: Adv Sci (Weinh). 2019 Apr 6;6(11):1900023. doi: 10.1002/advs.201900023 (PMC6548964; doi:10.1002/advs.201900023)
Supplement: Supplementary file 1 — Supplementary [file ADVS-6-1900023-s001.pdf]

Copyright WILEY-VCH Verlag GmbH & Co. KGaA, 69469 Weinheim, Germany, 2019.

Supporting Information

for *Adv. Sci.,* DOI: 10.1002/advs.201900023

Effective Delivery of Hypertrophic miRNA Inhibitor by Cholesterol-Containing Nanocarriers for Preventing Pressure Overload Induced Cardiac Hypertrophy

Ying Zhi, Chen Xu, Dandan Sui, Jie Du, Fu-Jian Xu,* and Yulin Li**

Supporting Information

Effective Delivery of Hypertrophic miRNA Inhibitor by Cholesterol-Containing Nanocarriers for Preventing Pressure Overload Induced Cardiac Hypertrophy

Ying Zhi,[†] *Chen Xu,*[†] *Dandan Sui, Jie Du,*[*] *Fu-Jian Xu*[*] *and Yulin Li*[*]

Y. Zhi, Prof. J. Du, Prof. Y. L. Li
Beijing Anzhen Hospital, Capital Medical University, The Key Laboratory of Remodeling-Related Cardiovascular Diseases, Ministry of Education, Beijing Institute of Heart Lung and Blood Vessel Diseases, Beijing 100029, China
Email: jiedu@ccmu.edu.cn (J. Du); lyllyl_1111@163.com (Y. L. Li)
C. Xu, Dr. D. Sui, Prof. F-J. Xu
Key Lab of Biomedical Materials of Natural Macromolecules (Beijing University of Chemical Technology), Ministry of Education, Beijing Laboratory of Biomedical Materials, Beijing Advanced Innovation Center for Soft Matter Science and Engineering, Beijing University of Chemical Technology, Beijing 100029, China
E-mail: xufj@mail.buct.edu.cn (F-J. Xu)

[†]Both authors contributed equally to this work.

Table S1 | PDI values of complexes

Samples	Average PDI[a] (at different N/P ratios)			
	**5**	**10**	**15**	**20**
CHO-PGEA/miRNA	0.17	0.16	0.15	0.13
PEI/miRNA	0.26	0.27	0.16	0.15

[a]Determined from DLS results (Source data are provided as a Source Data file). PDI = Polydispersity Index.

Table S2 | Primers used in this work.

Gene	Forward primer	Reverse primer
FOXO3	5'-CGTTGTTGGTTTGAATGTGG-3'	5'-GAGAGCAGATTTGGCAAAGG-3'
ANP	5'-GATTGGAGCCCAGAGTGGAC-3'	5'-CAGTGGCAATGTGACCAAGC-3'
GADPH	5'-CATGGCCTTCCGTGTTCCTA-3'	5'-GCGGCACGTCAGATCCA-3'

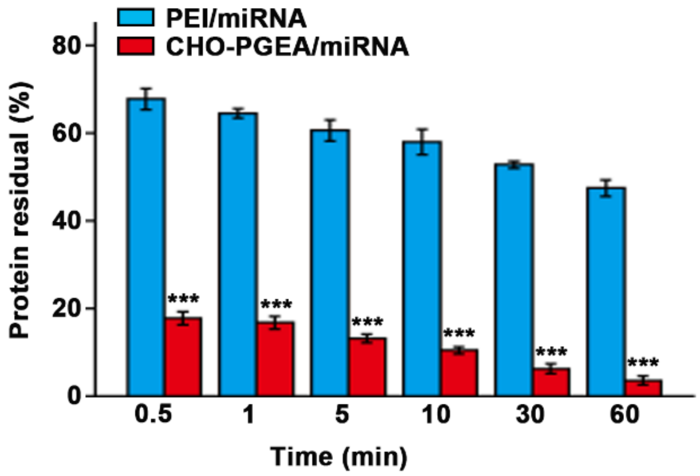

Figure S1. BSA protein assay of s-PGEA and PEI. Quantitative data are mean ± SEM, *** P<0.001 by Student's t-test (n = 3).

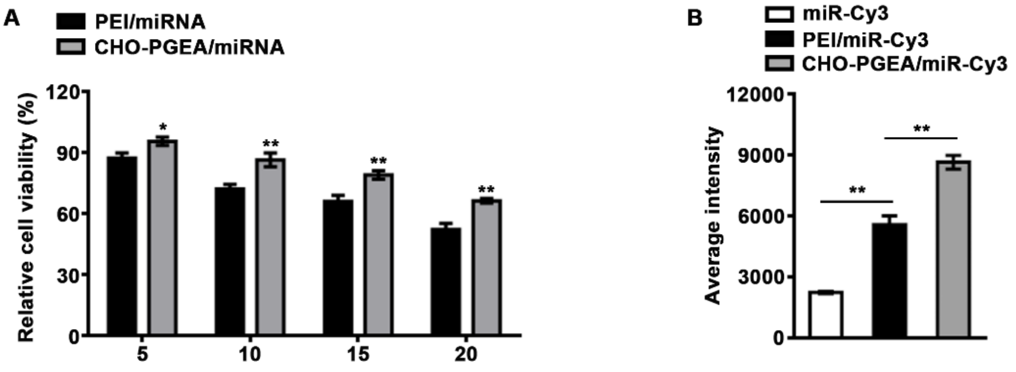

Figure S2. (**A**) MTT assay of H9C2 cells treated with CHO-PGEA/miRNA or PEI/miRNA complexes at various N/P ratios. (**B**) Average intensity of Cy3 in cellular internalization assay by MD. Quantitative data are mean ± SEM, **P*<0.05, ** *P*<0.01 by Student's t-test (n = 6).

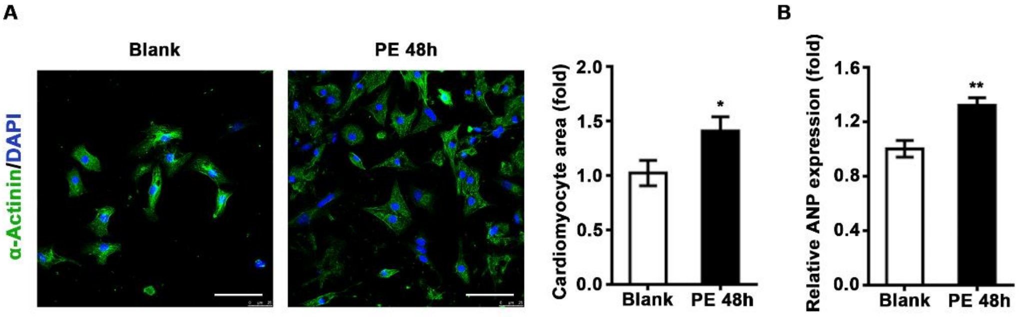

Figure S3. Hypertrophic cardiomyocytes induced by PE. (**A**) Confocal fluorescence images of immunofluorescence staining and quantification of cardiomyocyte areas (α-Actinin showed in green, and the nucleus is stained blue by DAPI; scale bar: 50 μm). (**B**) Relative expression levels of ANP. Quantitative data are mean ± SEM, *P<0.05, ** P<0.01 by Student's t-test (n = 4).
